# Intrinsic Functional Connectivity in Salience and Default Mode Networks and Aberrant Social Processes in Youth at Ultra-High Risk for Psychosis

**DOI:** 10.1371/journal.pone.0134936

**Published:** 2015-08-07

**Authors:** Andrea Pelletier-Baldelli, Jessica A. Bernard, Vijay A. Mittal

**Affiliations:** 1 Department of Psychology and Neuroscience, University of Colorado Boulder, Boulder, Colorado, United States of America; 2 Center for Neuroscience, University of Colorado Boulder, Boulder, Colorado, United States of America; 3 Department of Psychology, Texas A&M University, College Station, Texas, United States of America; 4 Department of Psychology, Northwestern University, Evanston, Illinois, United States of America; Institute of Psychology, Chinese Academy of Sciences, CHINA

## Abstract

Social processes are key to navigating the world, and investigating their underlying mechanisms and cognitive architecture can aid in understanding disease states such as schizophrenia, where social processes are highly impacted. Evidence suggests that social processes are impaired in individuals at ultra high-risk for the development of psychosis (UHR). Understanding these phenomena in UHR youth may clarify disease etiology and social processes in a period that is characterized by significantly fewer confounds than schizophrenia. Furthermore, understanding social processing deficits in this population will help explain these processes in healthy individuals. The current study examined resting state connectivity of the salience (SN) and default mode networks (DMN) in association with facial emotion recognition (FER), an integral aspect of social processes, as well as broader social functioning (SF) in UHR individuals and healthy controls. Consistent with the existing literature, UHR youth were impaired in FER and SF when compared with controls. In the UHR group, we found increased connectivity between the SN and the medial prefrontal cortex, an area of the DMN relative to controls. In UHR youth, the DMN exhibited both positive and negative correlations with the somatosensory cortex/cerebellum and precuneus, respectively, which was linked with better FER performance. For SF, results showed that sensory processing links with the SN might be important in allowing for better SF for both groups, but especially in controls where sensory processing is likely to be unimpaired. These findings clarify how social processing deficits may manifest in psychosis, and underscore the importance of SN and DMN connectivity for social processing more generally.

## Introduction

Understanding social domains of functioning is vital to any interpretation of human behavior. Specifically, knowing how social factors impact, interact, and sub-serve functioning must be understood in order to fully grasp how to make sense of the full range of human experience, including psychopathology. The National Institute of Mental Health (NIMH) Research Domain Criteria (RDoC) initiative emphasizes that clarifying the nature of social processes (i.e. social behavior and the neurobiological substrates that underlie this behavior) may be particularly informative to our understanding of human behavior and psychopathology [1]. In particular, one particular construct of social processes, social communication (i.e., affect/facial recognition, eye contact, production of facial expressions [1]), warrants attention, as these basic skills are integral and closely tied to social functioning (SF) (e.g., ability to maintain age appropriate relationships and limit interpersonal conflict).

Social deficits are a hallmark characteristic of schizophrenia (SZ), and recent evidence suggests that impairment is present prior to illness onset [2, 3]. Individuals who exhibit clinical symptoms indicative of this early stage, the psychosis prodrome, show subclinical levels of delusions, perceptual abnormalities, etc. and are defined as being at ultra high-risk (UHR) for the development of psychosis. Investigating UHR youth could aid in our understanding of etiological markers for schizophrenia [4], and identify new areas for intervention and preventative treatment. The evaluation of social processes in populations that show social communicative impairment (i.e. UHR) may lead to continued clarification of this domain of functioning. Further, research findings indicate that deficits in SF are one of the most prominent indicators of risk for psychosis [5], and clarifying how social processes manifest in UHR individuals could highlight areas for preventative treatment. In sum, due to the critical role social processes play in adolescent/young adult real-world functioning and the potential impact of evaluating UHR, there is increasing focus on studying social communication in UHR youth [6–8].

Of particular interest is the way in which UHR youth readily identify the emotional expressions of faces (facial emotion recognition; FER). The prevailing evidence suggests that FER deficits are already present in the prodromal stage of psychosis, as UHR youth consistently perform more poorly than their healthy peers on tasks that assess their ability to determine what emotional expression is being presented [9]. Such impairment is relevant as it is often linked with real-world SF. Further, changes in SF are some of the most commonly reported concerns among at-risk study participants and their families [10], and are a key factor in determining prognosis for at risk youth [11]. SF is also associated with core features of psychosis such as negative symptoms and cognition [12], suggesting it to be reflective of a central underlying pathology. In sum, both FER and SF appear to be target areas for research that could improve our knowledge surrounding the impaired functioning that is common in psychosis.

Understanding the neural underpinnings behind FER and SF is important because this knowledge aids in clarifying *why* a specific behavioral deficit is present. Such insight could aid in garnering information surrounding the specific mechanisms behind impairment, which may help our understanding of etiology and in turn, how to target treatments more effectively. In addition, it may provide insight into the component cognitive processes that underlie SF broadly. There is a growing body of evidence describing the neural basis of both FER and SF. The brain regions involved in FER in healthy individuals is well researched, and include a variety of cortical and subcortical areas [13–15]. However, insula activation is consistently evident when healthy individuals view facial emotion expressions [14]. The insula is a key hub in the brain’s salience network (SN) [16], along with regions of the anterior prefrontal cortex and anterior cingulate cortex [17, 18]. The SN is involved in determining the salience of environmental stimuli by recruiting sensory processing regions, and then allowing further action to be taken and/or limiting the involvement of other networks as needed [18, 19]. The role of the SN is therefore likely to be important for understanding salient emotional stimuli and social processes more broadly.

At present, research in psychosis has increased its focus on the SN, particularly through honing in on the insula. Studies examining connectivity within the SN show that decreased connectivity within the SN is evident in patients diagnosed with schizophrenia [18]. Furthermore, the available literature involving schizophrenia suggests that increased inter-network connectivity involving the insula relates to better FER ability [20]. Generally, research indicates that the insula is related to social processes [19]. No studies directly examining the SN and SF are currently available, although it has been implicated in other relevant clinical samples (e.g. autism spectrum disorders [21, 22]. In sum, the SN is becoming an increasingly recognized network in psychosis, and there is some evidence to suggest it is involved in social processes. Examining these links in UHR stands to aid in clarifying how any association between SN and FER/SF underlies the impairment that is commonly seen in this sample.

Studies suggest that another intrinsic network in the brain, the default mode network (DMN), may also be integral to social processes [23]. The DMN is comprised of a dorsal medial prefrontal cortex system along with regions located in the medial temporal lobe [24], and it is involved in internal cognition processes such as self-referential thinking and task independent thought [25–27]. Studies report mixed findings regarding the resting state connectivity of the DMN in schizophrenia, but in general, increased within network connectivity is the most consistent finding [23]. At present, the predominant method in clinical neuroscience is to examine connectivity within the DMN and social processes, with less focus on relationships with other brain regions. UHR research shows absent/reduced anticorrelations between the DMN and task-related networks in the brain relative to controls (significant anticorrelations are noted in healthy samples) [23, 28, 29]. Additionally, research in schizophrenia and related samples (e.g., autism spectrum disorder) suggests that stronger connectivity (or reduced negative correlations) between the DMN and other areas of the brain may result in poorer ability to switch between internal cognitive modes to perform a relevant task or social communication [23, 30, 31]. Furthermore, individuals with psychosis tend to exhibit a decreased ability to down-regulate activity in the DMN when presented with tasks [23], making this network particularly relevant for understanding how tasks may become impaired. Specifically, evidence shows that individuals with schizophrenia are less able to deactivate both DMN and FER regions while viewing faces, which is interpreted as likely indicating the perception of threat [32]. Research in non-clinical populations also highlights the DMN as a key factor in social processes [33]. Thus, this network may prove useful in aiding our understanding of mechanisms underlying the social communication deficits seen in UHR.

In accordance with a multiple levels of analysis RDoC approach, the current investigation utilized both behavior (FER and SF) and resting state functional connectivity of the SN and DMN to assess three primary aims. From a behavioral standpoint, the first aim was to examine FER and SF in UHR youth and controls. Both social variables were included in order to use a behavioral (FER) and clinical interview (SF) measure to evaluate social processes (in accordance with an attempt to use differing levels of analyses). Also, while we expect the neural circuitry underlying these two domains to be related [18, 20, 23], they are separate constructs measured in unique ways, and thus will likely generate different results. We predicted impairment in the UHR group across both domains [2, 8]. Secondly, we hypothesized that network connectivity involving the SN and DMN would differ across groups. Specifically, we expected that the UHR group would show decreased connectivity within the SN and increased connectivity within the DMN relative to controls [18, 23]. With respect to behavior, we hypothesized that less connectivity involving the SN and other brain regions, and higher connectivity concerning the DMN and other areas of the brain, would relate to poorer FER ability and SF, and that the nature of this relationship would be discrepant between groups [20, 30]. It is the interactions between these two networks and other brain regions that are expected to drive behavior, as opposed to connectivity within the network itself.

## Materials and Methods

### Participants

Participants included 69 adolescents/young adults (36 UHR and 33 controls), aged 16–21 (mean age = 18.86, SD = 1.5), who were recruited to the University of Colorado Boulder’s Adolescent Development and Preventive Treatment (ADAPT) research program. Email, newspaper advertisements, Craigslist, and community referrals were used to recruit UHR participants. Control participants were recruited through flyers and newspaper announcements. Exclusion criteria for both groups included history of head injury, neurological disorder, any contraindications to the magnetic resonance imaging (MRI) environment (e.g. current pregnancy or metal in the body), and having a DSM-IV-TR Axis I psychotic disorder or substance dependence. The presence of a psychotic disorder in a first-degree relative or meeting for an Axis I disorder was exclusionary criteria for controls.

In regards to diagnoses, there was a large discrepancy between groups as to whether they had a current Axis I diagnosis due to sampling methodology. For the UHR group, a variety of diagnoses were given including mood disorders, anxiety disorders, and substance use disorders (although no one met criteria for substance dependence). This variety of Axis I diagnoses is typical of an UHR population [34]. Due to this diagnostic presentation of the UHR group, current medication usage was also discrepant. Data was generated as to whether the participant was currently taking a psychotropic medication (yes or no) to be used as a covariate in imaging analyses. The University of Colorado Boulder Institutional Review Board approved the protocol and written informed consent procedures for this investigation.

### Materials and Procedure

The Structured Interview for Prodromal Syndromes (SIPS) [35] was administered to detect the presence of a prodromal syndrome. Study participants met criteria for a prodromal syndrome in three possible ways: 1) Attenuated Positive Symptom Prodromal Syndrome (a score between 3 and 5 on the SIPS based on a scale from 0 to 6) and/or 2) decline in global functioning accompanying the presence of schizotypal personality disorder and age <19 and/or 3) Genetic Risk and Deterioration Prodromal Syndrome. The SIPS gauges distinct categories of prodromal symptoms including positive and negative symptom dimensions. A total score is generated for each domain, with higher scores corresponding to greater symptomatology. The Structured Clinical Interview for the Diagnostic and Statistical Manual was administered to determine the presence of psychosis and substance dependence exclusionary criteria (SCID-I) [36].

The Global Functioning Scale-Social (GFS:S) [37] was administered by trained graduate students to clinically assess for SF over the past month. This interview was developed for use with UHR adolescents/young adults and rates engagement with peers/relationships, conflict with peers, and intimate/family relationships [38]. The scale is rated by the clinician and scored from 1–10 (higher scores indicate better functioning). The training of clinical interviewers (who were advanced doctoral students) was conducted over a 2-month period, and inter-rater reliabilities exceeded the minimum study criterion of Kappa ≥ 0.80.

The Penn Emotion Recognition Task (ER-40) is a computerized test of facial emotional recognition [39]. Participants view 40 faces displaying one of five emotions (happy, sad, anger, fear, or neutral) and determine which emotion is being expressed. Each emotional expression is presented eight times, with balanced representations of gender and ethnicity. This behavioral task is used in a variety of different studies, shows good psychometric properties, and is completed in around five minutes [40]. The order of stimuli is randomized, but fixed across subjects. The maximum score is 40, with higher scores indicating better performance.

#### Image acquisition and processing

Structural images were acquired with a T1-weighted 3D magnetization prepared rapid gradient multi-echo sequence (MPRAGE; sagittal plane; repetition time [TR] = 2,530 ms; echo times [TE] = 1.64 ms, 3.5 ms, 5.36 ms, 7.22 ms, 9.08 ms; GRAPPA parallel imaging factor of 2; 1 mm^3^ isomorphic voxels, 192 interleaved slices; FOV = 256 mm; flip angle = 7°). Additionally, a five minute 34 second resting state blood-oxygen-level dependent (BOLD) scan was acquired with a T2-weighted echo-planar functional protocol (number of volumes = 165; TR = 2,000ms; TE = 29 ms; matrix size = 64 x 64 x 33; FA = 75°; 3.8 x 3.8 x 3.5 mm^3^ voxels; 33 slices; FOV = 240 mm). Participants were instructed to relax with their eyes closed during the resting state scan time. A turbo spin echo proton density (PD)/T2-weighted acquisition (TSE; axial oblique aligned with anterior commissure-posterior commissure line; TR = 3,720 ms; TE = 89 ms; GRAPPA parallel imaging factor of 2; FOV = 240 mm; flip ange: 120°; 0.9 x 0.9 mm^2^ voxels; 77 interleaved 1.5 mm slices) was generated to investigate incidental pathology. Studies indicate that the functional connectivity MRI (fcMRI) duration utilized in the present study provides equal power to longer scan times [41].

Data were preprocessed in FSL (v. 5; http://fsl.fmrib.ox.ac.uk/fsl), which involved motion correction, brain extraction, high-pass filtering (100 s), and spatial smoothing (6mm FWHM). Next, functional images were aligned to the MNI 2-mm brain template with a two-step procedure. First, the resting state scan was aligned to the high-resolution MPRAGE using a linear boundary-based registration method, which relies on white matter boundaries [42–44]. Second, the MPRAGE was nonlinearly aligned to the template and the two registrations were then combined to align the functional resting state scan to the template.

Recent papers have demonstrated the importance of properly correcting for motion by not only regressing out motion parameters, but also regressing out or eliminating specific frames with motion outliers [45]. To accomplish this, we used the Artifact Rejection Toolbox (ART; http://www.nitrc.org/projects/artifact_detect/) to create confound regressors for motion parameters (3 translation and 3 rotation parameters), and additional confound regressors for specific image frames with outliers based on brain activation and head movement. In order to identify outliers in brain activation, the mean global brain activity (i.e., the mean signal across all voxels) was calculated as a function of time, and was then Z normalized. Outliers were defined as any frames where the global mean signal exceeded 3 SD. Similarly, frame-wise measures of motion (composite measure of total motion across translation and rotation) were used to identify any motion outliers (i.e., motion spikes). Motion outliers were defined as any frame where the motion exceeded 1 mm.

All functional connectivity analyses were performed in the CONN toolbox 14.p [46], with SPM8 (Wellcome Department of Imaging Neuroscience, London, UK; www.fil.ion.ucl.ac.uk/spm). Anatomical images were segmented into gray matter, white matter, and CSF with SPM8 in order to create masks for signal extraction. The CONN toolbox [46] uses principal component analysis (PCA) to extract 5 temporal components from the segmented CSF and white matter, which were entered as confound regressors in the subject-level GLM. This approach corrects for confounds of motion and physiological noise without regressing out global signal, which has been shown to introduce spurious anticorrelations [47, 48]. Motion from the ART toolbox was included as a confound regressor. From the motion translation parameters, the ART toolbox calculates mean displacement, and we used this measure as well as the number of motion and mean signal outliers in order to compare the degree of head movement between the groups. Further preprocessing included a band-pass filter (0.008 to 0.09 Hz), detrending, and despiking, in accordance with procedures used to target resting state data [46]. The mean time-series, averaged across all voxels within each seed was used as a regression parameter, and correlated with all other voxel in the brain in seed-to-voxel connectivity analyses.

#### Imaging analysis

**Fig 1:**
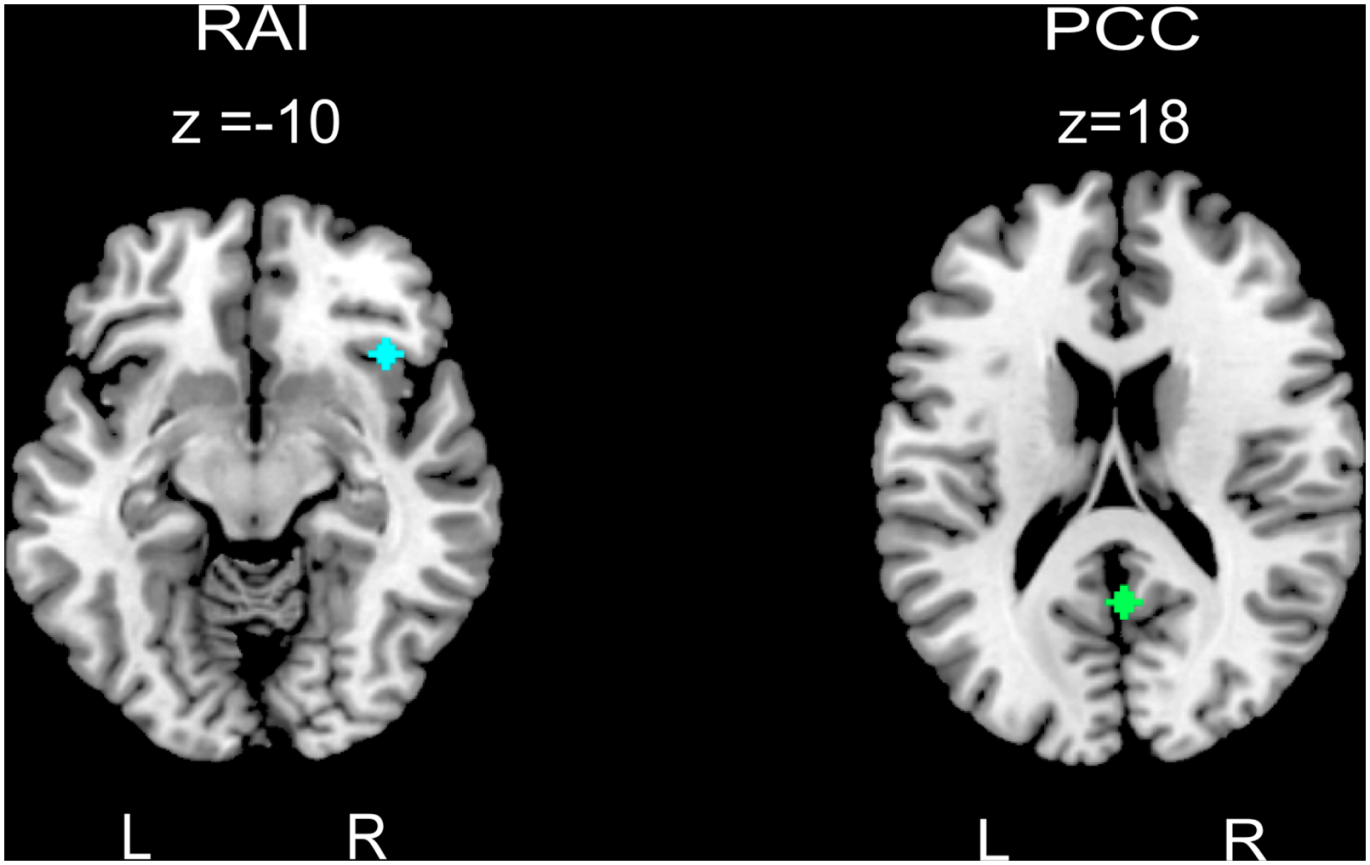
Seed regions of interest. *Note*: The salience network, defined using a seed in the rAI (right anterior insula), MNI coordinates (38, -22, 10). The default mode network, defined using a seed in the PCC (posterior cingulate cortex), MNI coordinates (1, -55, 17).

Functional connectivity was performed in the conn toolbox v. 14p [46]. Both the SN and DMN were evaluated using resting state functional connectivity using seed-to-voxel connectivity across the whole brain. Resting-state functional connectivity allows for the identification of specific intrinsic functional networks without the potential confounds of introducing functional tasks [23]. A priori seeds for these networks were masked based on established literature and single seeds (as opposed to multiple) were used to represent nodes of the SN and DMN in order to limit analyses and maximize power [16, 49] (Fig 1). The right anterior insula (rAI) is a crucial node of the SN [18] and was used to define the SN network, while the posterior cingulate cortex (PCC) represented the node for the DMN, consistent with previous UHR research [28, 29]. Seeds were generated using the Wake Forest University PickAtlas [50, 51] using a 4mm radius.

First, we confirmed that the pattern of connectivity of the SN and DMN in our control and UHR sample was in line with established research [18, 23] (see S1–S4 Tables). Due to the impact of medication on the brain [52, 53], all between group analyses and UHR within group analyses controlled for whether a participant was currently taking a psychotropic medication or not. Following our inspection of the SN and DMN in both groups, we conducted between group analyses examining differences in both networks using seed to voxel connectivity across the whole brain. Next, we investigated relationships between SN and DMN connectivity in regards to both FER and SF, again using seed-to-voxel connectivity in the whole brain. In order to evaluate the relationship between connectivity and behavior, we examined connectivity involving the SN and DMN with both FER and SF within each group separately along with testing whether an interaction was present whereby the relationship between connectivity and behavior differed based on group status. Within network connections were defined as associations among a priori seeds and voxels of regions clearly established by literature to comprise either the SN (e.g. anterior cingulate cortex) or DMN (e.g. medial prefrontal cortex). In contrast, hypotheses evaluating connectivity of the SN/DMN and other brain regions examined associations among a priori seeds and voxels in the brain that do not belong to regions that typically comprise either the SN or DMN. Results of all analyses were thresholded at the voxel-level at *p*_*uncorrected*_ <0.001 and then corrected at the cluster-level using a false-discovery rate (FDR) of *p*<0.05 [54].

#### Statistical analysis

ANOVA models were used to evaluate group differences in demographic variables, FER, and SF. Because preliminary studies examining social processes impairment have found effect sizes at or exceeding 0.31 [55], there is good evidence to suggest that the current sample has adequate power to detect the hypothesized relationship. Further, the present evaluation exceeds the existing UHR literature sample sizes where evaluation of the SN and DMN has taken place [28, 29] along with the one study to examine familial risk for psychosis, the DMN, and social functioning [30]. Based on existing research, the current sample is adequately powered to detect group differences in social processes, connectivity, and links between connectivity and behavior.

## Results

### Demographics and Symptom Characteristics

There were no significant group differences in age (F(1,68) = 0.90, *p* = 0.345), sex (F(1,68) = 2.79, *p* = 0.100), and parental education (F(1,68) = 2.35, *p* = 0.130). Based on the sampling strategy for the controls (they could not meet current criteria for an DSM-IV Axis I diagnosis), there were significant differences between groups in regards to whether participants were currently taking a psychotropic medication (F(1,68) = 108.37, *p* < .001) (Table 1).

**Table 1:** Sample characteristics.

	Ultra High Risk (n = 36)	Control (n = 34)
%Male	69.40	50.00
Age	19.03 (1.4)	18.68 (1.7)
Parental Education (years)	16.37 (1.9)	15.46 (2.9)
SIPS Positive**	12.11 (4.4)	0.47 (1.3)
SIPS Negative**	10.06 (6.5)	0.50 (0.9)
Total ER-40 performance*	31.39 (3.4)	33.03 (2.2)
Social Functioning**	6.44 (1.6)	8.65 (0.8)
%Psychotropic Medication**	47.18	2.94
%antipsychotics	8.30	0.00
%SSRIs	19.44	0.00
%stimulants	19.44	2.94
%Current Axis I diagnosis**	69.40	0.00
Imaging Outliers*	6.54(4.6)	3.94(5.1)
Imaging Motion	0.24(0.2)	0.26(0.4)

Unless otherwise indicated, values are mean (standard deviation). SIPS (Structured Interview for Prodromal Syndromes); ER-40 (Emotion Recognition 40 Task); Social functioning represented score on the Global Functioning Scale: Social (GFS:S). Current Axis I diagnosis is based off DSM-IV criteria. Medications are shown indicating the percentage of the sample taking the medication.

*p<0.05

**p<0.001)

### Facial emotion recognition and social functioning

UHR participants performed significantly worse than controls on the FER task (F(1,68) = 5.62, *p* = 0.011, β = 0.82; the β value refers to the regression coefficient estimating the relationship between group and FER performance and gives information regarding directionality). UHR youth also showed significant impairment on SF when compared to controls (F(1,68) = 51.21, *p* < 0.001, β = 1.10) (Table 1).

### Group differences in the SN and DMN

**Fig 2:**
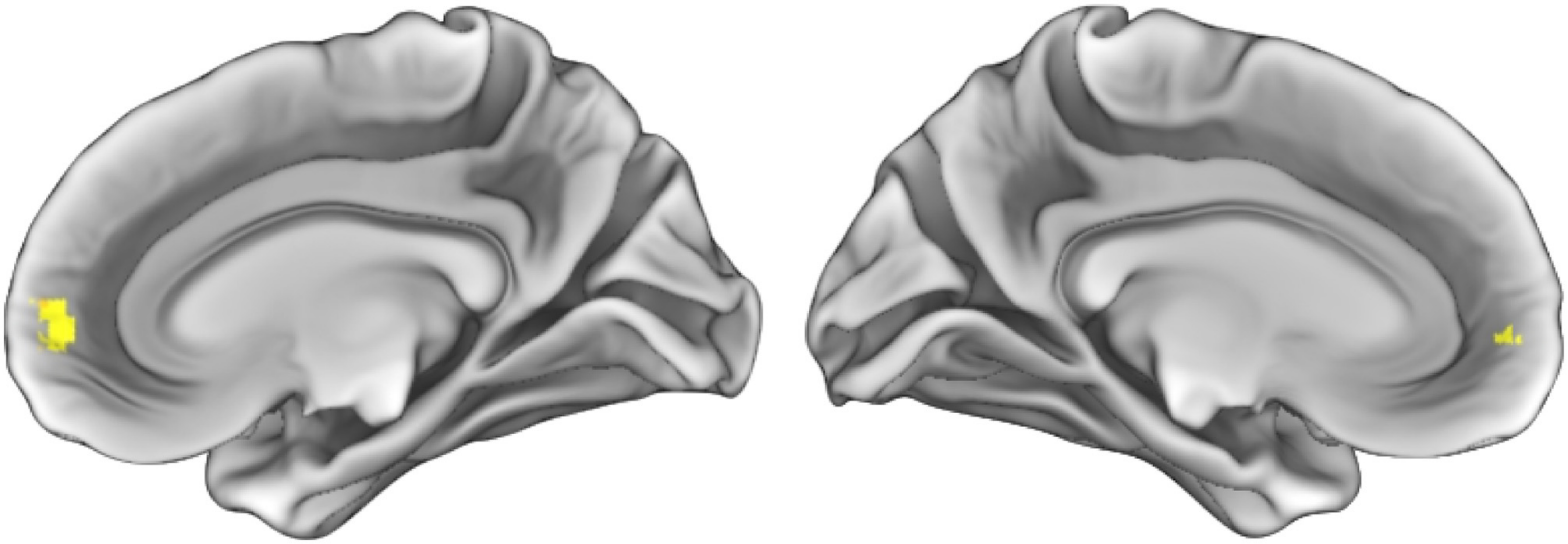
Group Differences in seed to voxel connectivity. *Note*: Results show increased connectivity between the salience network (as defined by the right anterior insula seed region) and the medial prefrontal cortex in ultra high-risk youth relative to controls. Results are thresholded at the voxel-level at p_uncorrected_ <0.001 and then corrected at the cluster-level using a false-discovery rate (FDR) of *p*<0.05.

Independent t-tests were performed to investigate group differences in total motion and outliers. Results show significant group differences in the number of outliers, (*t* = 2.23, *p* = 0.03), but not in motion (*t* = -0.35, *p* = 0.73), and both were included as covariates in analyses. In contrast to expectations, there were no group connectivity differences within the SN network itself; rather, UHR youth showed increased connectivity between the SN and the medial prefrontal cortex (mPFC) relative to controls. Thus, there was increased connectivity between the SN and a key node in the DMN. There were no significant group differences in the DMN as defined using the PCC, which was in contrast to expected results (Fig 2, Table 2)

**Table 2:** Resting state functional connectivity: regions and coordinates.

Group differences in seed to voxel connectivity involving the salience network						
			MNI Coordinates			
Region	BA	Cluster Size	x	Y	z	*t*-Value
Right Frontal Pole	10	179	10	56	2	4.50
Associations between facial emotion recognition and connectivity involving the default mode network in ultra-high risk youth						
			MNI Coordinates			
Region	BA	Cluster Size	x	y	z	*t*-Value
Left Postcentral Gryus	3	769	-42	-32	60	5.09
Right Precentral Gyrus	3	631	48	-22	60	5.39
Right Cerebellum	N/A	320	14	-58	-18	5.68
*Precuneus	7	491	-6	-60	50	6.05
Associations between social functioning and connectivity involving the salience network						
			MNI Coordinates			
Region	BA	Cluster Size	x	y	z	*t*-Value
Left Cuneal Cortex	18	168	-6	-82	30	3.77

*Note*. The salience network was defined by the right anterior insula (rAI) as a seed, while the default mode network was defined using the posterior cingulate cortex (PCC). Findings show that UHR youth showed greater connectivity between the rAI and right frontal pole cluster in comparison with controls. Secondly, among the UHR group, positive correlations involving the postcentral gyrus, precentral gyrus, and cerebellum was evident along with an inverse relationship of connectivity among the PCC and precuneus. Finally, positive connectivity with the cuneal cortex and the rAI was more evident for controls, while an inverse relationship was evident with this same region for UHR individuals. Results of all analyses were thresholded at the voxel-level at p_uncorrected_ <0.001 and then corrected at the cluster-level using a false-discovery rate (FDR) of p<0.05.

* denotes an inverse relationship of signal.

### Associations between connectivity, facial emotion recognition, and social functioning

**Fig 3:**
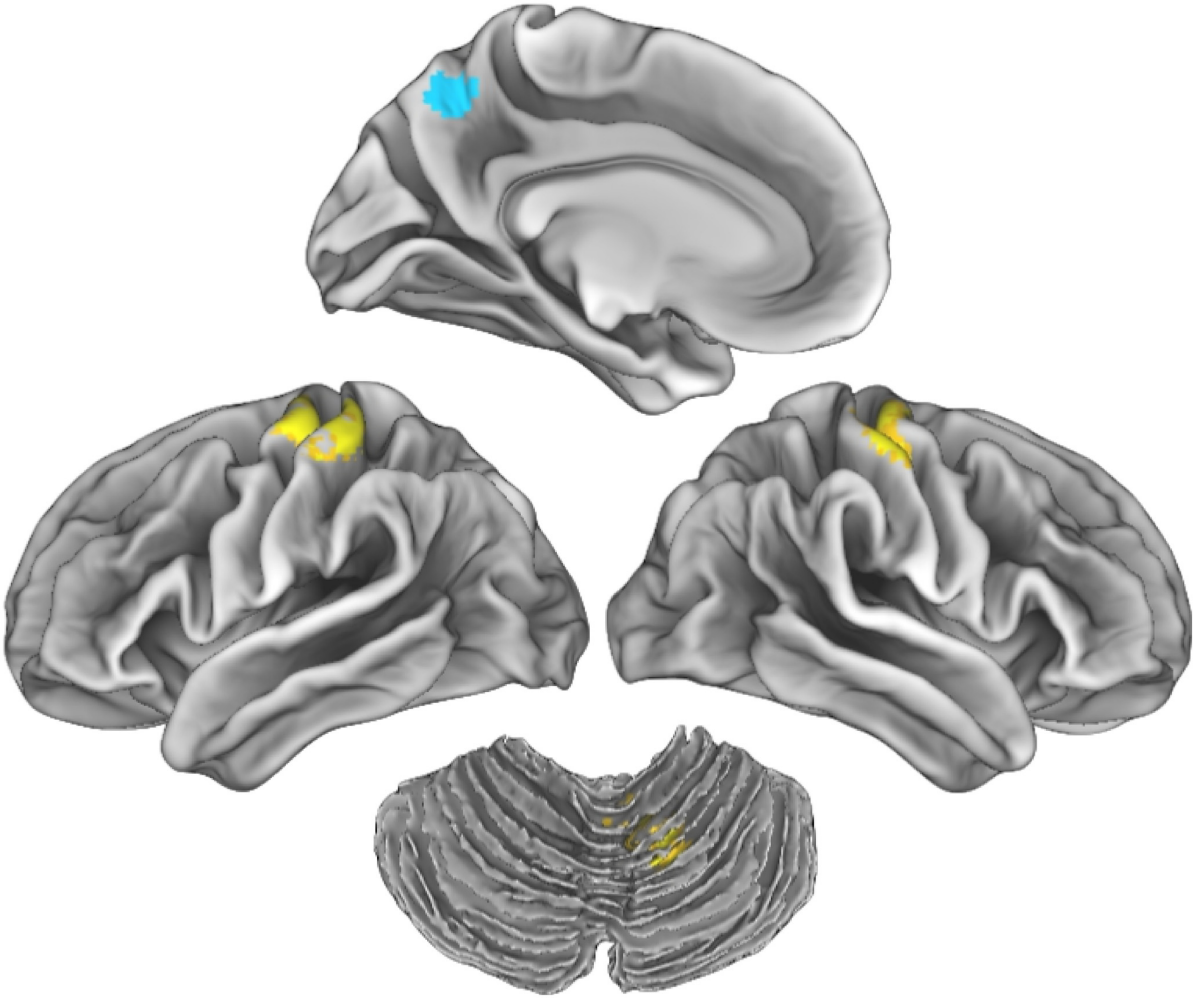
Associations between connectivity and facial emotion recognition. *Note*: Results indicate positive correlations (yellow) between the default mode network (defined by the posterior cingulate cortex as a seed region) and the somatosensory cortex (shown bilaterally) and the cerebellum among UHR individuals. An inverse relationship in signal (blue) between the default mode network with the precuneus is shown in the medial left hemisphere at the top. Results are thresholded at the voxel-level at *p*_*uncorrected*_ <0.001 and then corrected at the cluster-level using a false-discovery rate (FDR) of *p*<0.05.

**Fig 4:**
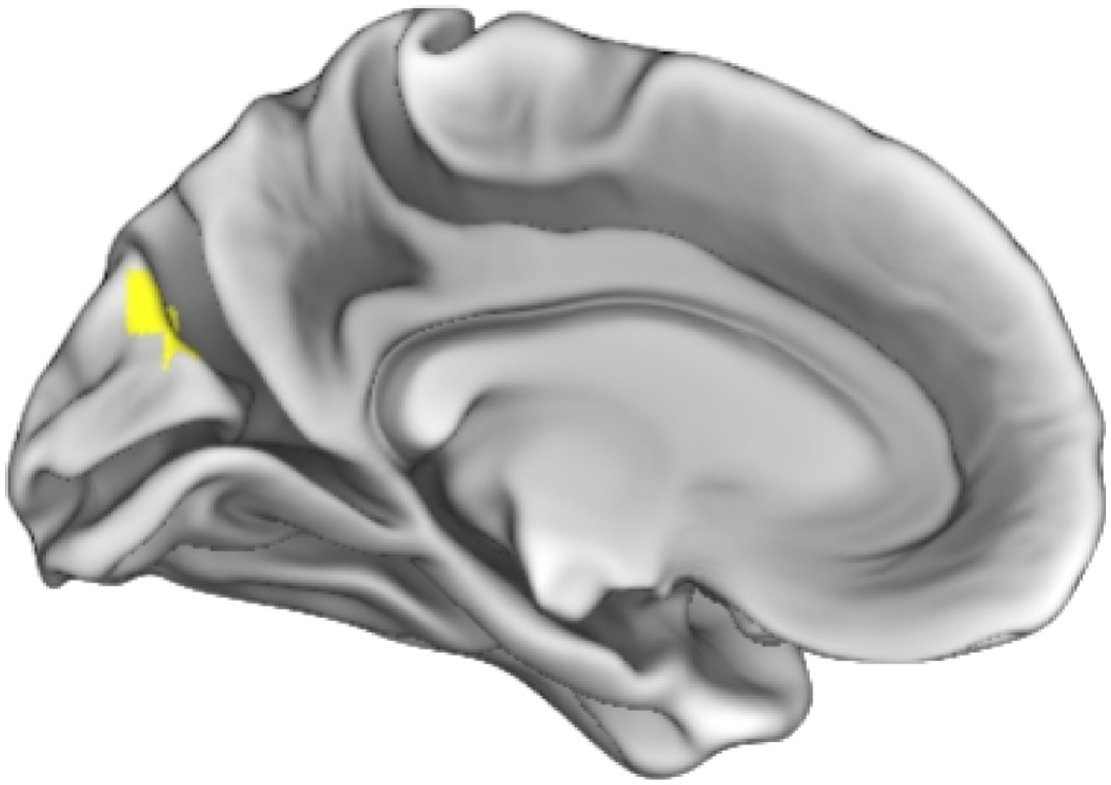
Associations between connectivity and social functioning. *Note*: Results show a positive correlation between signal of the salience network (defined by the right anterior insula) and the occipital cortex. This relationship was different for controls and ultra-high risk youth (UHR). Connectivity associations were positive for the control group (shown above) and negative for UHR individuals. Results are thresholded at the voxel-level at p_uncorrected_ <0.001 and then corrected at the cluster-level using a false-discovery rate (FDR) of *p*<0.05.

Contrary to our hypotheses, the SN did not show any significant associations with FER. However, findings linking the DMN and FER were evident. Higher correlations of the resting state signal between the PCC and both the cerebellum and the somatosensory cortex was linked with better ability to recognize emotional expressions in UHR youth, which was in contrast to the expected direction. However, as expected, lower connectivity between the PCC and precuneus was associated with better FER ability, also in UHR youth. Results relating the SN and SF were also consistent with our predictions. There was a significant interaction whereby positive connectivity between the SN (represented by the rAI) and cuneal cortex related to higher SF scores for controls, and an inverse relationship between SN signal and cuneal cortex was linked with higher SF performance for UHR youth. There were no significant associations between the DMN and SF, which was contrary to our hypotheses (Figs 3 and 4, Table 2, S5–S8 Figs).

## Discussion

To our knowledge, this is the first study to examine FER, SF, and intrinsic functional connectivity in UHR youth. Not only do our findings provide important new insights with regards to social processing deficits in UHR individuals and psychosis more broadly, but we also identified connectivity-behavior associations that point to cognitive processes (e.g., bottom-up sensory integration, internal mentation, mental simulation) that are important for normative social processes. This neuropsychological approach has highlighted the importance of these networks and cognitive contributions, and has increased our understanding of both FER and SF. We provide behavioral evidence consistent with prior literature indicating impairments in both FER and SF in UHR individuals [2, 8, 38, 55, 56] and extend these findings by demonstrating that performance in UHR individuals is associated with intrinsic network connectivity. These findings further clarify the mechanisms underlying social process deficits and in turn, point to specific connections within the brain that are relevant for psychosis development. We discuss these findings with respect to the larger literature in turn below.

### Group differences in the DMN and SN

Consistent with prior research [29], UHR showed increased connectivity between the SN and DMN in comparison with controls, as evidenced by connectivity between the SN and the mPFC (which is a region of the DMN). However, previous findings linked the rAI with the PCC, not the mPFC as shown in the present study [29]. Research suggests that increased activity of the DMN during tasks implies an impaired ability to shut down internal cognition and perhaps rumination in specific clinical populations [23]. One interpretation of this result is that increased connectivity between the DMN and SN in UHR relative to controls is due to an inability in UHR youth to effectively “shut down” the DMN when cued by the SN to attend to a relevant task. In sum, these results imply that the functioning of the SN in determining the salience of environmental stimuli and engaging/disengaging other relevant networks [18] is different in UHR youth, even when accounting for the potential impact of medication. Furthermore, although our findings were with another node of the DMN (the mPFC), they indicate that SN-DMN interactions are altered in UHR youth.

There were no group differences DMN connectivity when seeded using the PCC. This result was not surprising, given the mixed nature of the extant literature. Despite some UHR work showing disruptions in PCC connectivity [28, 29], the findings are mixed, with other work showing no group differences in PCC connectivity in those at familial high-risk and controls [57] Similarly, investigations involving individuals diagnosed with schizophrenia are also inconsistent. Repovs and colleagues [49] found no group differences between patients and controls, while Rotarska-Jagiela and colleagues [58] found decreased connectivity in patients. Although Whitfield-Gabrilei and Ford [23] conclude in their review that increased within network connectivity is found more often in the DMN in schizophrenia, the authors do acknowledge the mixed results. Incongruent results may be due to a variety of factors, such as sample size (the present investigation contained a larger sample than the other UHR studies), UHR symptom severity across samples, analysis pipeline, cluster correction threshold, and/or variation in whether potential medication confounds were taken into account. DMN connectivity results are widely varied both in the psychosis risk samples and patients with schizophrenia, and the present null result is not necessarily at odds with the current literature in the field. It is possible that the increased connectivity within network is not present until later in the developmental trajectory of psychosis.

### Associations between connectivity, facial emotion recognition, and social functioning

In examining the link between connectivity of the DMN and SN with FER, results emerged highlighting the role of DMN connectivity in UHR youth. First, a positive association in connectivity of the PCC and both the cerebellum and postcentral gyrus was linked to better FER performance in the UHR group, suggesting that the greater the connectivity between these regions, the better the FER ability within this sample. Further, lower connectivity between the PCC and precuneus was tied to higher FER behavioral scores. Although the cerebellum, postcentral gyrus, and precuneus are not typically associated with FER, research does indicate that these areas are engaged during the viewing of affective faces [13, 14]. The cerebellum, postcentral gyrus, and PCC are linked with facial emotion recognition tasks across all types of emotional expressions, while the precuneus is activated during the presentation of happy faces [13, 14]. Thus, connectivity between the PCC and these cortical regions is in support of existing FER research.

The specific direction of these connections suggests differential relationships with the DMN and FER ability. Generally, FER entails a sequence of events involving the perception of faces, construction of facial representations in the brain, followed by association and linking of emotional knowledge to perception [59]. The multiple regions involved in the present findings reflect this wide array of psychological activity that underlies facial emotion recognition [60]. As the DMN is involved in self-referential thought [27], the present bidirectional connectivity patterns may represent varying ways in which attention to internal states influence, and even impede this complex FER process.

Although the interactions among the many neural regions involved in FER are not yet fully understood [14], some hypotheses can be generated regarding the nature of the present results. First, the direction of the association between the PCC and postcentral gyrus/cerebellum finding is in contrast to our expected results. There is research to suggest that negative correlations between the DMN and other brain regions at rest may relate to improved cognition and social behavior [23, 30, 31]. As such, it is notable that the present finding represents the opposite direction of activity. To our knowledge, this is the first investigation of the DMN and FER in UHR youth. It may be that a unique direction of connectivity exists in regard to this specific FER task. One interpretation of this conflicting result involves the simulation theory of FER, where an individual attempts to simulate the emotional expression seen in another in order to facilitate recognition of that emotion [59]. The postcentral gyrus represents the somatosensory cortex, and it may be that stronger connectivity between this specific region and the DMN allow for better down-regulating of the DMN in favor of recruiting regions needed to simulate emotion [13]. Similarly, the cerebellar lobule V was also implicated in our analyses. This region of the cerebellum is associated with motor processing [61–63] and may be part of the network required for facial simulation. Further, the cerebellum has also been implicated more generally in emotion processing [64]. These findings provide additional support for this simulation theory of emotion recognition, and underscore the importance of intact networks for simulation and network interactions in FER.

The present investigation showed that less within-DMN connectivity (PCC-precuneus) is associated with better FER performance in UHR youth. This finding is contradictory to the results presented above. The precuneus is a region of the DMN (albeit not a core hub; [23, 24] and is involved in the processing of internal information [65]. Aberrant increased connectivity within the DMN is related to psychosis symptoms [66], suggesting some disruption of internal processes in schizophrenia. Thus, the UHR individuals with lower within-DMN connectivity show a more normative connectivity pattern and fewer symptoms resulting in better FER performance.

It is somewhat surprising that there were no results involving connectivity of the SN in association with either group in regard to FER. In light of the purported role of the SN in evaluating stimuli and recruiting regions needed to generate a reaction and response [18], we had hypothesized that connectivity in the SN would be related to FER. The lack of results in the present study is in contrast with a recent meta-analyses showing that the insula is consistently activated when viewing facial emotions [14]. However, unlike the current investigation, the meta-analysis solely evaluated task related activation, which is a different method than the resting state connectivity examined here. These different methods may contribute to the disparate findings noted above. Similarly, there were no findings involving the control group in regard to FER, either in regard to the DMN (as in UHR) or the SN. It is possible that our control group did not have enough variability in their social communicative ability (mean = 33.03, range = 28–37, SD = 2.2) compared to UHR group (mean = 31.39, range = 25–36, SD = 3.4), and therefore, we were unable to detect any associations that may exist.

Although FER did not relate to the SN, there were significant associations between SF and SN connectivity. The connectivity relationships with SF differed based on group. Better SF ability was linked with greater connectivity between the SN and the visual cortex for controls, while the opposite direction of connectivity was evident in the UHR group. This result supports research indicating that the SN is involved in sensory information processing [18]. Notably, another study showed that there is a reduction of influence of the visual cortex on the insula in individuals diagnosed with schizophrenia or schizoaffective disorder, suggesting that this aberrant relationship relates to dysfunction in bottom-up processes [67]. For example, if an individual were unable to visually process information correctly (e.g., someone’s facial expressions or voice tone), this would then lead to problems in higher cognitive processes (such as interpretation of social cues) and would likely contribute to social functioning impairment [68]. In regard to the positive relationship between connectivity and SF for controls, the present investigation shows that a negative association of signal between these two regions (perhaps indicative of irregular bottom-up processing) does appear to manifest in poorer social functioning. However, it is more challenging to interpret how less connectivity between the SN and visual cortex is better for SF for UHR youth. It is possible that UHR youth, who may exhibit risk for impaired sensory processing as seen in schizophrenia[68], rely less on this region for SF relative to controls. In total, our results suggest that connectivity between the SN and the visual cortex is important for maintaining good SF, although the directions of these connectivity patterns are discrepant among groups.

Finally, it is worth noting that there were no behavioral associations with connectivity involving the mPFC, though both FER and SF were impaired relative to controls and mPFC connectivity with the SN was disrupted in UHR youth. The SN-mPFC link was the only association that showed significant group differences when examining resting state connectivity alone, but the mPFC was not involved in either the FER task or the SF measure. Given that no behavioral relationship was evident with the mPFC, it could be interpreted that there are actually no FER or SF consequences of exhibiting abnormally increased connectivity between the SN and the mPFC. This explanation would be somewhat surprising, as it is believed that the SN attends to salient stimuli in the environment and cues the DMN to deactivate in order to move forward on a particular task [69, 70]. Further, research shows that damage to the white matter tract connecting the rAI and DMN results in an impaired ability to down regulate the DMN activity [71]. On the other hand, it is possible that other measures, such as self-evaluation/theory of mind or even tasks that require less self-referential thought, could tap into this group difference in connectivity involving the mPFC more directly. The current evaluation does highlight that when a clinical sample exhibits a differential pattern of connectivity, it does not necessarily equate to detrimental behavioral consequences. Thus, while the mPFC and SN were not associated with behavioral performance, our results point to several additional network and patterns of connectivity that do explain the variability in FER and SF.

### Limitations

Several limitations to the present investigation should be acknowledged. First, the control sample did not include those with an Axis I clinical diagnoses. While this aided in our ability to maximize power to determine group differences, future studies would benefit from having a more continuous comparison group that would be more representative of the general population. This more heterogeneous sample would also allow for future work that could focus on the specificity of these results to UHR youth relative to other clinical samples. Further, while the FER task utilized in the present study is widely researched in UHR and SZ, it lacks ecological validity (e.g. voice and body cues); future work would benefit from such paradigms. Finally, as in all studies of functional connectivity, analyses of connectivity are correlational and do not necessarily reflect causation.

### Conclusions

Consistent with the RDoC initiative, the present study evaluated social processes from multiple levels of analysis, and the results improve our understanding of how social impairment may arise in psychosis. In regards to FER, greater connectivity involving the DMN and FER regions related to better ability to recognize faces in UHR youth. It is possible that these connections with FER regions and the DMN network allow for improved regulation for task efficiency and performance, but future research should evaluate this hypothesis more directly as it conflicts with some of the existing literature. In contrast, an inverse relationship of connectivity within the DMN was associated with better FER performance in UHR youth. Finally, better SF was linked with connections between the SN and visual cortex for both groups, but was more apparent in controls. This result may represent a failure in bottom-up processing manifesting in behavioral social impairment [66–67].

In sum, the present study highlights multiple networks in the brain that relate to both healthy and impaired social processes. In support of research linking the DMN with social processes [33], it appears that DMN is especially important for FER performance in UHR youth. Further, initial sensory processing ability aids in one’s ability to function socially, and apeears to be especially important for healthy individuals. Not only does this work provide important new targets for research and remediation in UHR youth, but it also highlights the significance of intact component cognitive processes (e.g., emotion simulation, internal mentation, and bottom-up processing) to successful social processing.

## Supporting Information

S1 Fig Salience Network Connectivity in Controls. *Note*: Connectivity involving the salience network was represented by analyzing seed to voxel connectivity of the right anterior insula. Results of all analyses were thresholded at the voxel-level at p_uncorrected_ <0.001 and then corrected at the cluster-level using a false-discovery rate (FDR) of p<0.05.
(TIFF)

S2 Fig Salience Network Connectivity in UHR. *Note*: Connectivity involving the salience network was represented by analyzing seed to voxel connectivity of the right anterior insula. Results of all analyses were thresholded at the voxel-level at p_uncorrected_ <0.001 and then corrected at the cluster-level using a false-discovery rate (FDR) of p<0.05.
(TIFF)

S3 Fig Default Mode Network Connectivity in Controls. *Note*: Connectivity involving the default mode network was represented by analyzing seed to voxel connectivity of the posterior cingulate cortex. Results of all analyses were thresholded at the voxel-level at p_uncorrected_ <0.001 and then corrected at the cluster-level using a false-discovery rate (FDR) of p<0.05.
(TIFF)

S4 Fig Default Mode Network Connectivity in UHR. *Note*: Connectivity involving the default mode network was represented by analyzing seed to voxel connectivity of the posterior cingulate cortex. Results of all analyses were thresholded at the voxel-level at p_uncorrected_ <0.001 and then corrected at the cluster-level using a false-discovery rate (FDR) of p<0.05.
(TIFF)

S5 Fig Correlations between connectivity of the posterior cingulate cortex and left somatosensory cortex with facial emotion recognition performance. *Note*: PCC (posterior cingulate cortex); ER-40 (Emotion Recognition Task). Data presented is for visual purposes only and represents associations between connectivity and total performance on the ER-40 task. Higher ER-40 totals represent better performance. Results of all connectivity analyses were thresholded at the voxel-level at p_uncorrected_ <0.001 and then corrected at the cluster-level using a false-discovery rate (FDR) of p<0.05.
(TIFF)

S6 Fig Correlations between connectivity of the posterior cingulate cortex and right somatosensory cortex with facial emotion recognition performance. *Note*: PCC (posterior cingulate cortex); ER-40 (Emotion Recognition Task). Data presented is for visual purposes only and represents associations between connectivity and total performance on the ER-40 task. Higher ER-40 totals represent better performance. Results of all connectivity analyses were thresholded at the voxel-level at p_uncorrected_ <0.001 and then corrected at the cluster-level using a false-discovery rate (FDR) of p<0.05.
(TIFF)

S7 Fig Correlations between connectivity of the posterior cingulate cortex and precuneus with facial emotion recognition performance. *Note*: PCC (posterior cingulate cortex); ER-40 (Emotion Recognition Task). Data presented is for visual purposes only and represents associations between connectivity and total performance on the ER-40 task. Higher ER-40 totals represent better performance. Results of all connectivity analyses were thresholded at the voxel-level at p_uncorrected_ <0.001 and then corrected at the cluster-level using a false-discovery rate (FDR) of p<0.05.
(TIFF)

S8 Fig The relationship between connectivity and social functioning differs between groups. *Note*: rAI (right anterior insula); social functioning is measured by the Global Functioning Scale: Social whereby higher scores represent better overall current social functioning). Data presented is for visual purposes only and represents associations between connectivity and social functioning based on group status (UHR: ultra high risk; CTRL: control). Results of all connectivity analyses were thresholded at the voxel-level at p_uncorrected_ <0.001 and then corrected at the cluster-level using a false-discovery rate (FDR) of p<0.05.
(TIFF)

S1 Table Salience Network Connectivity in Controls. *Note*: * denotes negative correlation, otherwise positive correlations are indicated. Connectivity involving the salience network was represented by analyzing seed to voxel connectivity of the right anterior insula. Results of all analyses were thresholded at the voxel-level at p_uncorrected_ <0.001 and then corrected at the cluster-level using a false-discovery rate (FDR) of p<0.05.
(DOCX)

S2 Table Salience Network Connectivity in UHR. *Note*: * denotes negative correlation, otherwise positive correlations are indicated. Connectivity involving the salience network was represented by analyzing seed to voxel connectivity of the right anterior insula. Results of all analyses were thresholded at the voxel-level at p_uncorrected_ <0.001 and then corrected at the cluster-level using a false-discovery rate (FDR) of p<0.05.
(DOCX)

S3 Table Default Mode Network Connectivity in Controls. *Note*: * denotes negative correlation, otherwise positive correlations are indicated. Connectivity involving the default mode network was represented by analyzing seed to voxel connectivity of the posterior cingulate cortex. Results of all analyses were thresholded at the voxel-level at p_uncorrected_ <0.001 and then corrected at the cluster-level using a false-discovery rate (FDR) of p<0.05.
(DOCX)

S4 Table Default Mode Network Connectivity in UHR. *Note*: * denotes negative correlation, otherwise positive correlations are indicated. Connectivity involving the default mode network was represented by analyzing seed to voxel connectivity of the posterior cingulate cortex. Results of all analyses were thresholded at the voxel-level at p_uncorrected_ <0.001 and then corrected at the cluster-level using a false-discovery rate (FDR) of p<0.05.
(DOCX)
