# Supplementary figures and images for: Intrinsic Functional Connectivity in Salience and Default Mode Networks and Aberrant Social Processes in Youth at Ultra-High Risk for Psychosis
Source: PLoS One. 2015 Aug 7;10(8):e0134936. doi: 10.1371/journal.pone.0134936 (PMC4529226; doi:10.1371/journal.pone.0134936)

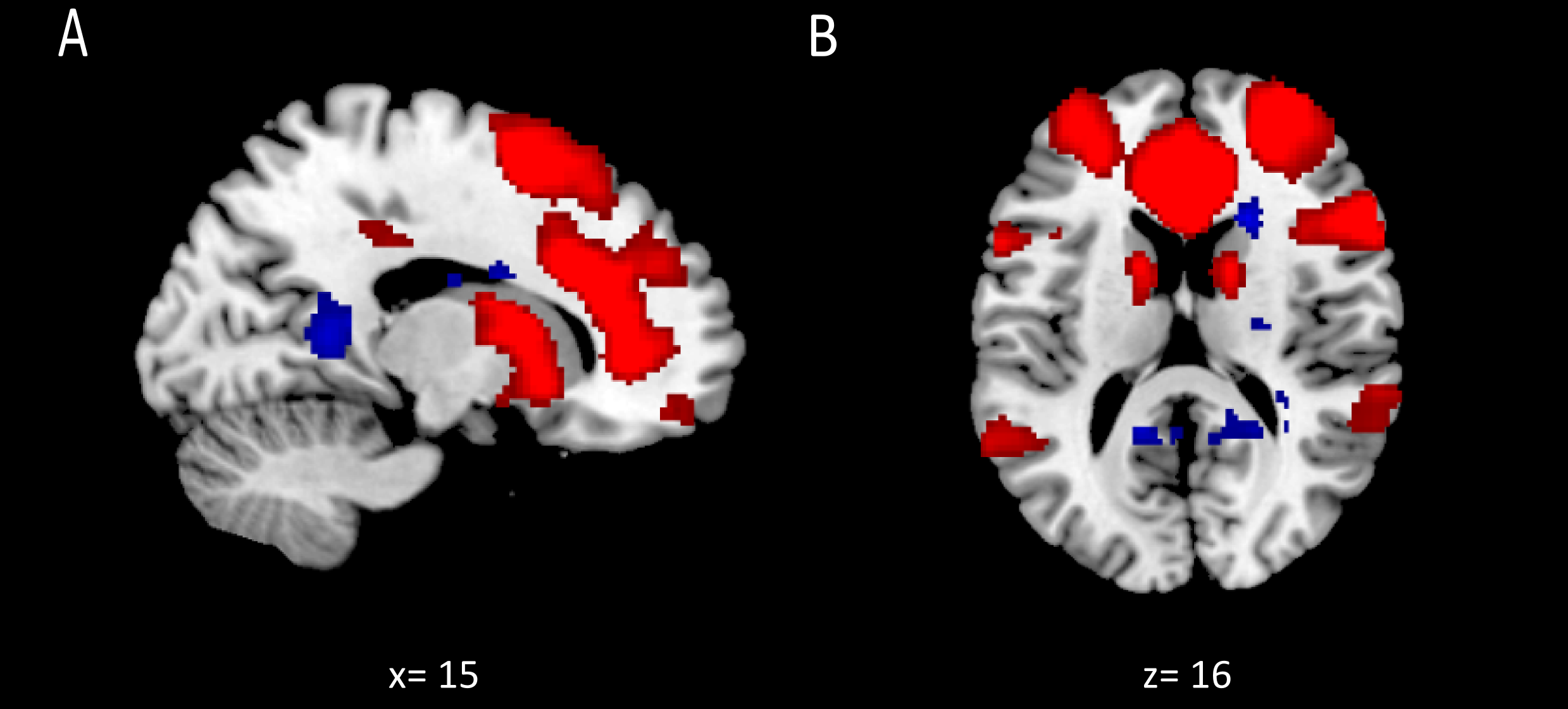

Supplement: S1 Fig — Note: Connectivity involving the salience network was represented by analyzing seed to voxel connectivity of the right anterior insula. Results of all analyses were thresholded at the voxel-level at puncorrected <0.001 and then corrected at the cluster-level using a false-discovery rate (FDR) of p<0.05. (TIFF) [file pone.0134936.s001.tiff]

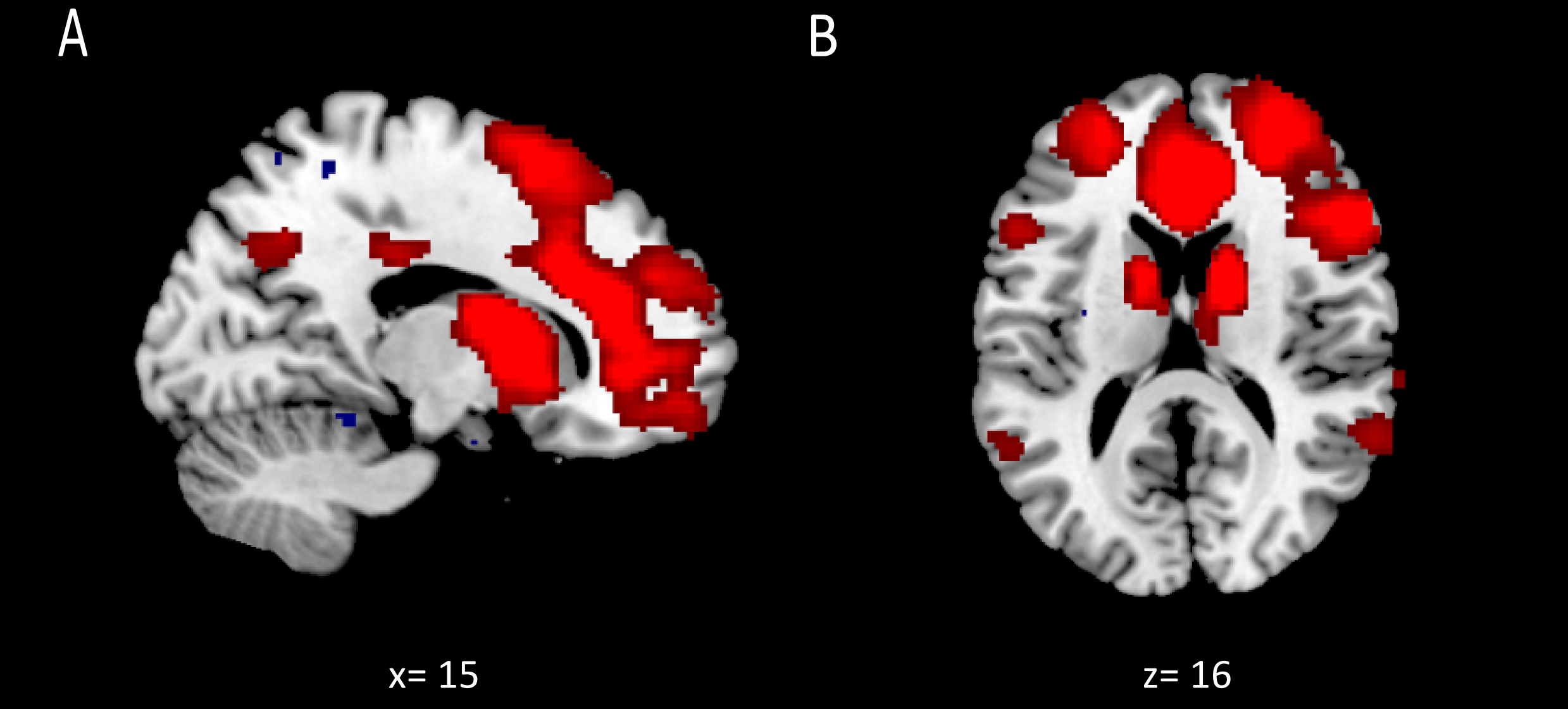

Supplement: S2 Fig — Note: Connectivity involving the salience network was represented by analyzing seed to voxel connectivity of the right anterior insula. Results of all analyses were thresholded at the voxel-level at puncorrected <0.001 and then corrected at the cluster-level using a false-discovery rate (FDR) of p<0.05. (TIFF) [file pone.0134936.s002.tiff]

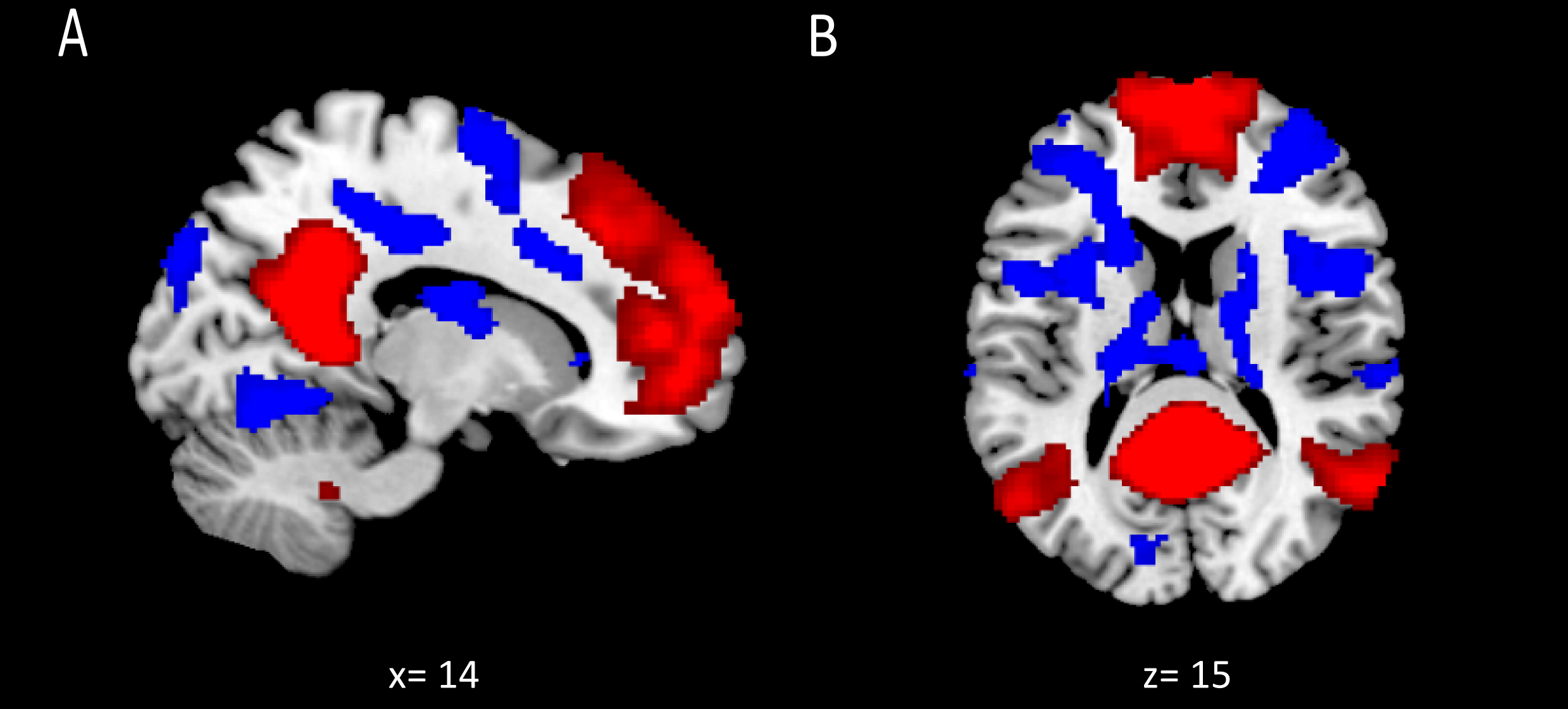

Supplement: S3 Fig — Note: Connectivity involving the default mode network was represented by analyzing seed to voxel connectivity of the posterior cingulate cortex. Results of all analyses were thresholded at the voxel-level at puncorrected <0.001 and then corrected at the cluster-level using a false-discovery rate (FDR) of p<0.05. (TIFF) [file pone.0134936.s003.tiff]

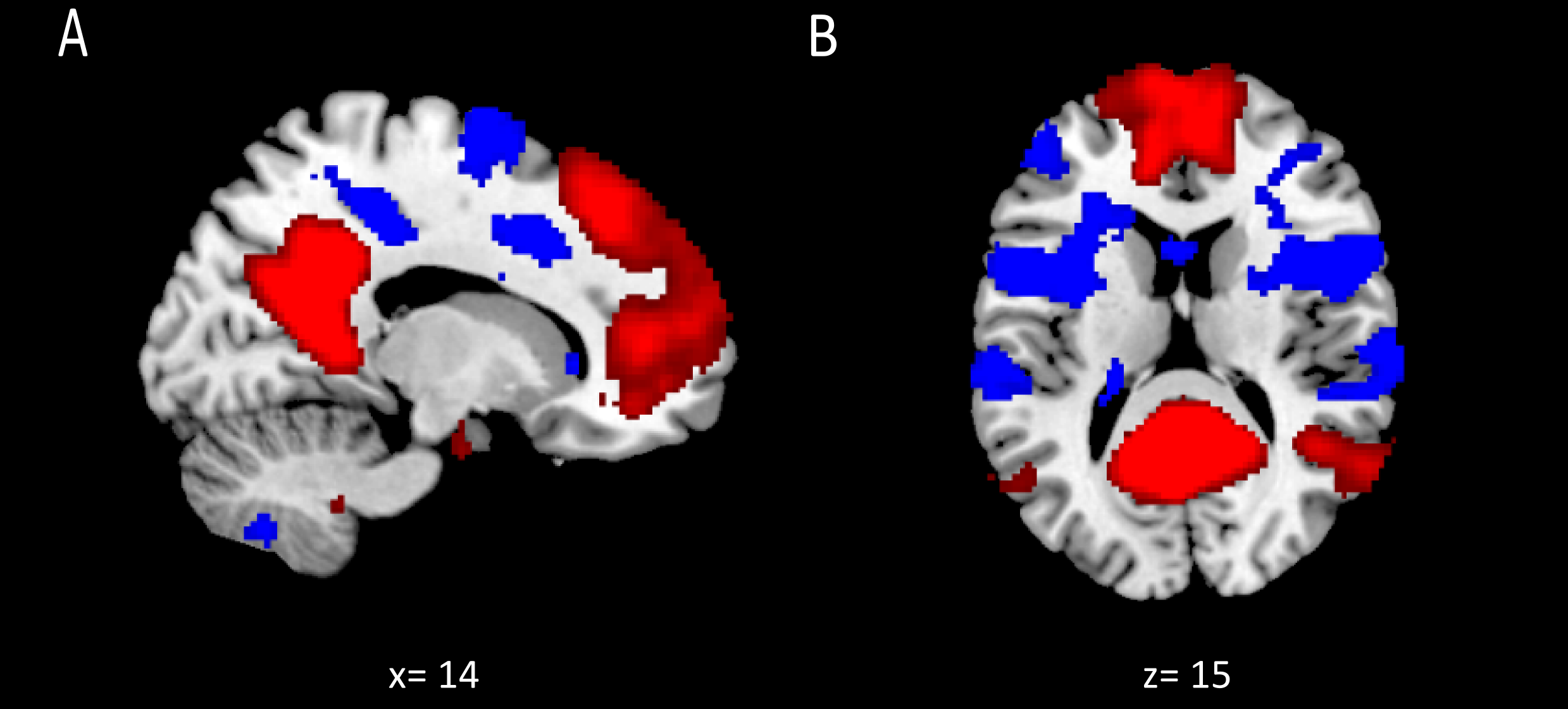

Supplement: S4 Fig — Note: Connectivity involving the default mode network was represented by analyzing seed to voxel connectivity of the posterior cingulate cortex. Results of all analyses were thresholded at the voxel-level at puncorrected <0.001 and then corrected at the cluster-level using a false-discovery rate (FDR) of p<0.05. (TIFF) [file pone.0134936.s004.tiff]

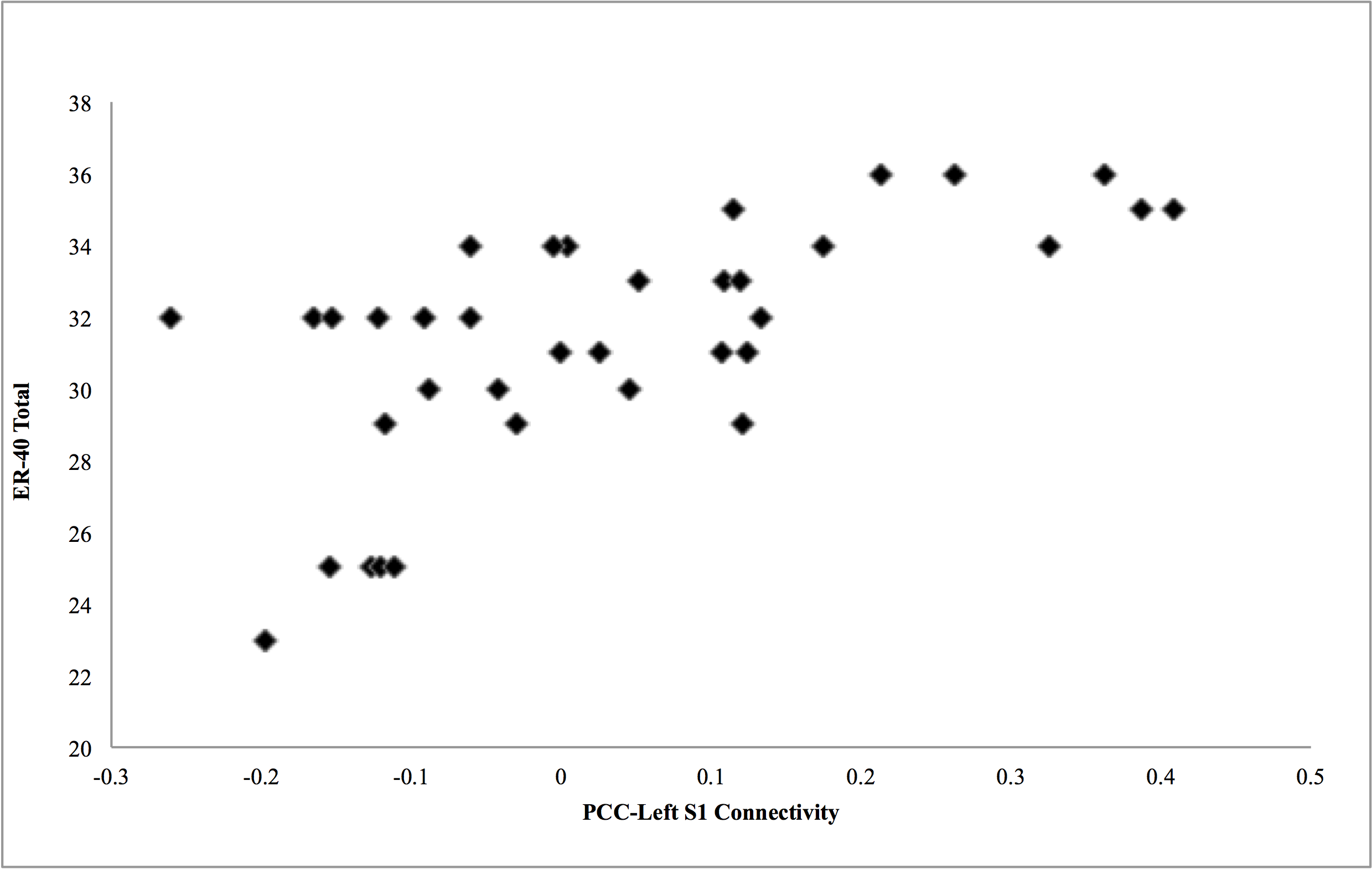

Supplement: S5 Fig — Note: PCC (posterior cingulate cortex); ER-40 (Emotion Recognition Task). Data presented is for visual purposes only and represents associations between connectivity and total performance on the ER-40 task. Higher ER-40 totals represent better performance. Results of all connectivity analyses were thresholded at the voxel-level at puncorrected <0.001 and then corrected at the cluster-level using a false-discovery rate (FDR) of p<0.05. (TIFF) [file pone.0134936.s005.tiff]

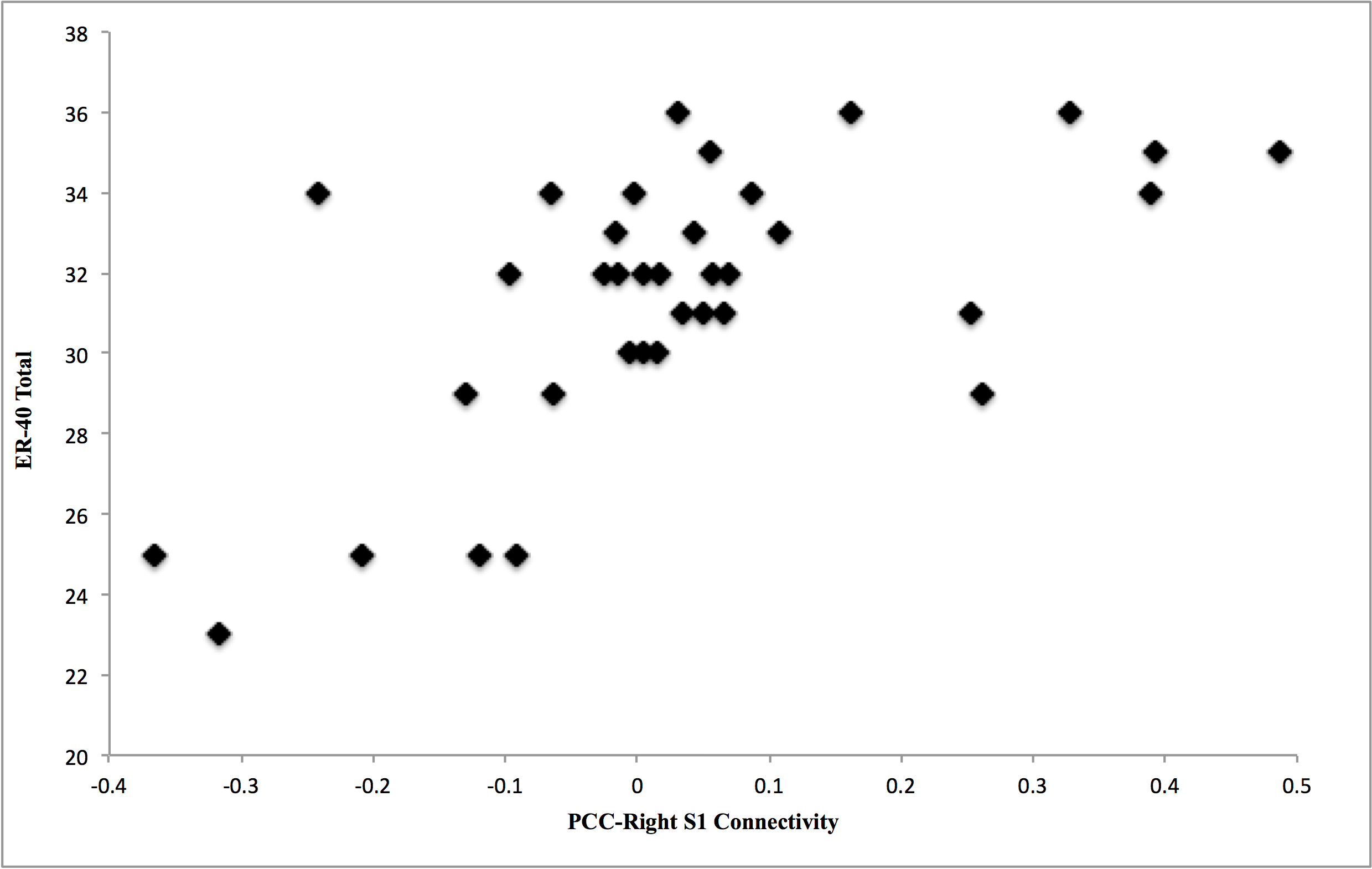

Supplement: S6 Fig — Note: PCC (posterior cingulate cortex); ER-40 (Emotion Recognition Task). Data presented is for visual purposes only and represents associations between connectivity and total performance on the ER-40 task. Higher ER-40 totals represent better performance. Results of all connectivity analyses were thresholded at the voxel-level at puncorrected <0.001 and then corrected at the cluster-level using a false-discovery rate (FDR) of p<0.05. (TIFF) [file pone.0134936.s006.tiff]

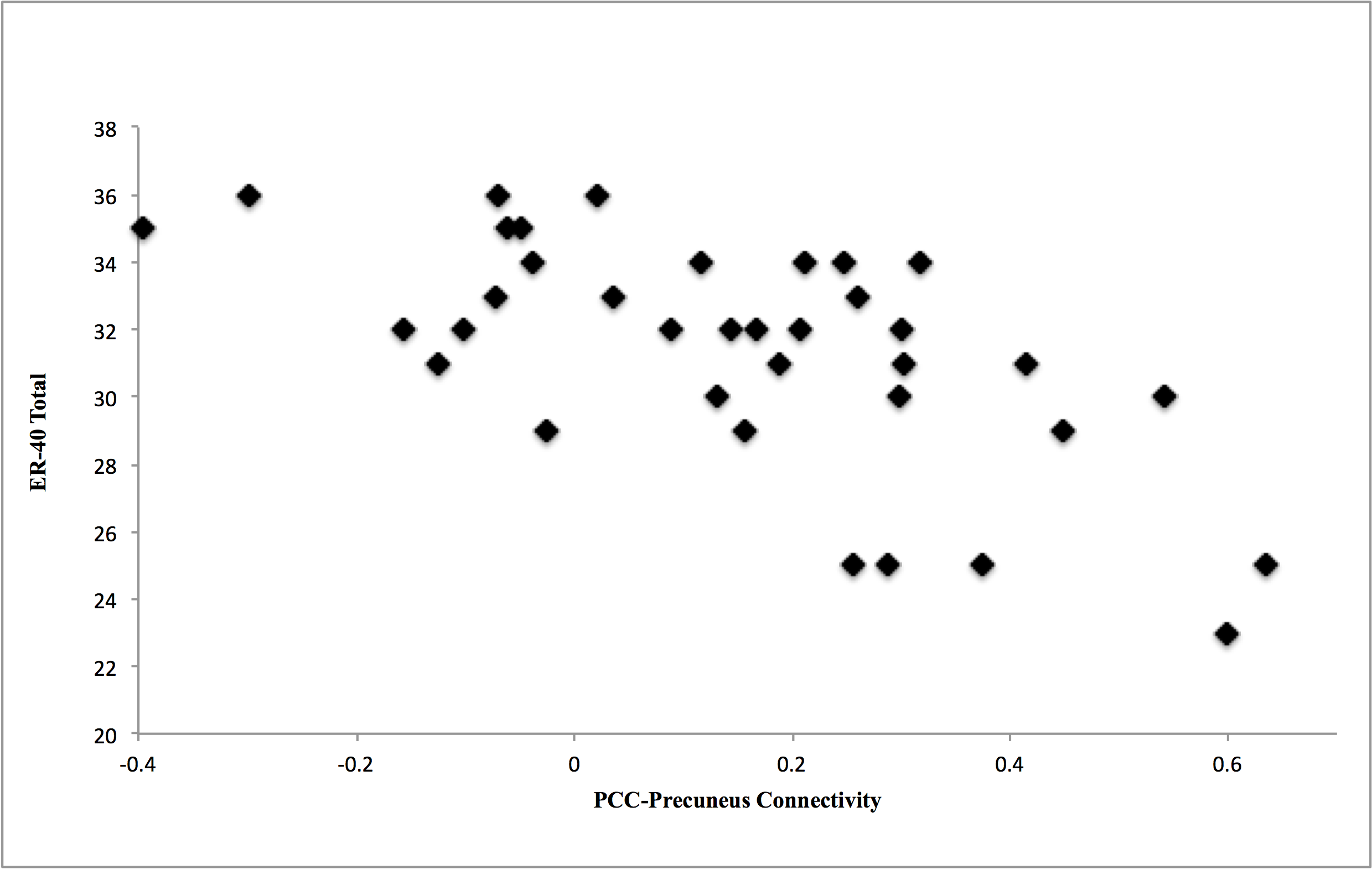

Supplement: S7 Fig — Note: PCC (posterior cingulate cortex); ER-40 (Emotion Recognition Task). Data presented is for visual purposes only and represents associations between connectivity and total performance on the ER-40 task. Higher ER-40 totals represent better performance. Results of all connectivity analyses were thresholded at the voxel-level at puncorrected <0.001 and then corrected at the cluster-level using a false-discovery rate (FDR) of p<0.05. (TIFF) [file pone.0134936.s007.tiff]

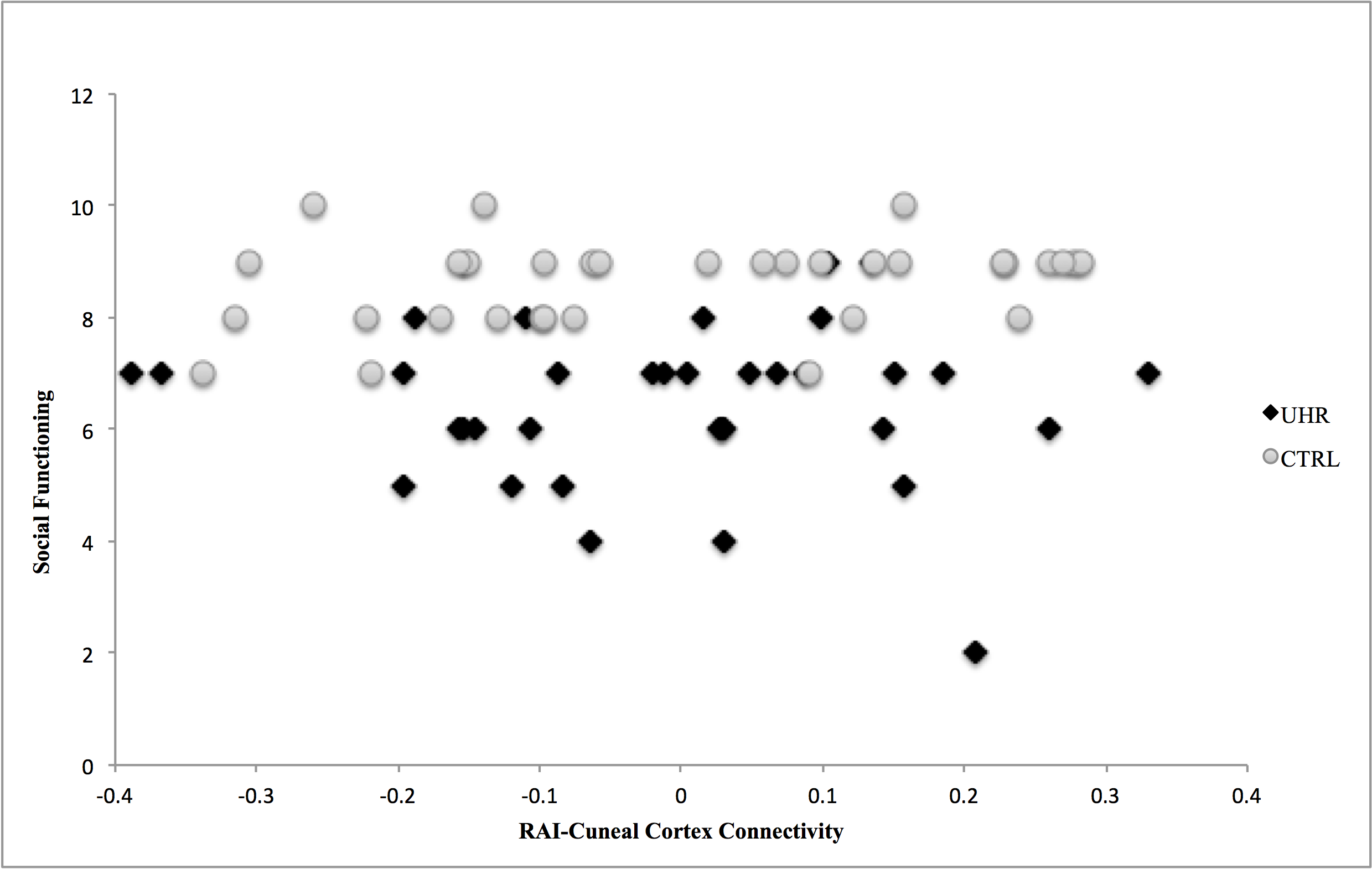

Supplement: S8 Fig — Note: rAI (right anterior insula); social functioning is measured by the Global Functioning Scale: Social whereby higher scores represent better overall current social functioning). Data presented is for visual purposes only and represents associations between connectivity and social functioning based on group status (UHR: ultra high risk; CTRL: control). Results of all connectivity analyses were thresholded at the voxel-level at puncorrected <0.001 and then corrected at the cluster-level using a false-discovery rate (FDR) of p<0.05. (TIFF) [file pone.0134936.s008.tiff]
