# Supplementary material for: Intrinsic Functional Connectivity in Salience and Default Mode Networks and Aberrant Social Processes in Youth at Ultra-High Risk for Psychosis
Source: PLoS One. 2015 Aug 7;10(8):e0134936. doi: 10.1371/journal.pone.0134936 (PMC4529226; doi:10.1371/journal.pone.0134936)
Supplement: S1 Table — Note: * denotes negative correlation, otherwise positive correlations are indicated. Connectivity involving the salience network was represented by analyzing seed to voxel connectivity of the right anterior insula. Results of all analyses were thresholded at the voxel-level at puncorrected <0.001 and then corrected at the cluster-level using a false-discovery rate (FDR) of p<0.05. (DOCX) [file pone.0134936.s009.docx]

**S1 Table. Salience Network Connectivity in Controls**

|  |  |  | MNI Coordinates | | |  |
| --- | --- | --- | --- | --- | --- | --- |
| Region | BA | Cluster Size | x | y | z | *t*-Value |
| Right Frontal Pole | 47 | 24804 | 38 | 24 | -10 | 50.28 |
| Right Supramarginal Gyrus | 40 | 4253 | 56 | -46 | 36 | 11.01 |
| Left Insular Cortex | 47 | 3937 | -38 | 18 | -10 | 21.15 |
| Left Supramarginal Gyrus | 40 | 1677 | -56 | -48 | 36 | 8.04 |
| Posterior Cingulate Gyrus | 23 | 1586 | 6 | -20 | 40 | 8.31 |
| Left Crus1 of the Cerebellum | N/A | 696 | -42 | -50 | -42 | 6.37 |
| Left Temporal Gyrus | 20 | 279 | -60 | -28 | -10 | 5.70 |
| *Precuneus | 30 | 730 | -4 | -52 | 6 | 5.56 |
| *Medial Temporal lobe | 48 | 623 | 20 | 10 | 26 | 6.38 |
| *Left Amygdala | N/A | 203 | -24 | -12 | -14 | 4.82 |
| *Middle Temporal lobe | 37 | 159 | 36 | -44 | 8 | 5.22 |
| *Left V of the Cerebellum | N/A | 132 | -20 | -48 | -18 | 4.69 |

*Note:* ***** denotes negative correlation, otherwise positive correlations are indicated. Connectivity involving the salience network was represented by analyzing seed to voxel connectivity of the right anterior insula. Results of all analyses were thresholded at the voxel-level at p_uncorrected_ <0.001 and then corrected at the cluster-level using a false-discovery rate (FDR) of p<0.05
