# Supplementary material for: Intrinsic Functional Connectivity in Salience and Default Mode Networks and Aberrant Social Processes in Youth at Ultra-High Risk for Psychosis
Source: PLoS One. 2015 Aug 7;10(8):e0134936. doi: 10.1371/journal.pone.0134936 (PMC4529226; doi:10.1371/journal.pone.0134936)
Supplement: S2 Table — Note: * denotes negative correlation, otherwise positive correlations are indicated. Connectivity involving the salience network was represented by analyzing seed to voxel connectivity of the right anterior insula. Results of all analyses were thresholded at the voxel-level at puncorrected <0.001 and then corrected at the cluster-level using a false-discovery rate (FDR) of p<0.05. (DOCX) [file pone.0134936.s010.docx]

**S2 Table. Salience Network Connectivity in UHR**

|  |  |  | MNI Coordinates | | |  |
| --- | --- | --- | --- | --- | --- | --- |
| Region | BA | Cluster Size | x | y | z | *t*-Value |
| Right Frontal Pole | 47 | 39505 | 38 | 20 | -10 | 56.18 |
| Left Supramarginal Gyrus | 40 | 1416 | -56 | -46 | 38 | 10.37 |
| Left Crus 1 of the Cerebellum | N/A | 765 | -10 | -78 | -28 | 6.31 |
| Left Middle Temporal Gyrus | 21 | 412 | -60 | -30 | -6 | 5.42 |
| Right Temporal Pole | 20 | 253 | 46 | 12 | -44 | 5.97 |
| Precuneus Cortex |  | 187 | 14 | -60 | 40 | 5.21 |
| Brain Stem | N/A | 179 | 6 | -16 | -20 | 5.74 |
| Left Crus 2 of the Cerebellum | N/A | 134 | -30 | -80 | -44 | 5.68 |
| Left Cerebellum | N/A | 121 | -42 | -54 | -42 | 4.32 |
| Precuneus Cortex | 7 | 87 | -10 | -68 | 34 | 4.07 |
| *Left V of the Cerebellum | N/A | 948 | -18 | -44 | -16 | 5.60 |
| *Right Lingual Gyrus | 30 | 441 | 18 | -44 | -14 | 4.94 |
| *Left Pallidum | N/A | 328 | -22 | -14 | 0 | 7.90 |
| *Right Postcentral Gyrus | 4 | 318 | -22 | -32 | 78 | 4.92 |
| *Right Superior Parietal Lobule | 5 | 248 | 20 | -50 | 58 | 5.02 |
| *Left Hippocampus | N/A | 223 | -20 | -8 | -22 | 5.81 |
| *Right Opercular Cortex | 48 | 169 | -36 | -6 | 20 | 5.19 |
| *Right Temporal Fusiform Cortex | 36 | 151 | 38 | 2 | -28 | 5.44 |
| *Right Hippocampus | N/A | 132 | 22 | -10 | -22 | 5.64 |
| *Left Superior Parietal Lobule | 5 | 128 | -18 | -54 | 56 | 4.80 |

*Note:* ***** denotes negative correlation, otherwise positive correlations are indicated. Connectivity involving the salience network was represented by analyzing seed to voxel connectivity of the right anterior insula. Results of all analyses were thresholded at the voxel-level at p_uncorrected_ <0.001 and then corrected at the cluster-level using a false-discovery rate (FDR) of p<0.05
