# Supplementary material for: Intrinsic Functional Connectivity in Salience and Default Mode Networks and Aberrant Social Processes in Youth at Ultra-High Risk for Psychosis
Source: PLoS One. 2015 Aug 7;10(8):e0134936. doi: 10.1371/journal.pone.0134936 (PMC4529226; doi:10.1371/journal.pone.0134936)
Supplement: S3 Table — Note: * denotes negative correlation, otherwise positive correlations are indicated. Connectivity involving the default mode network was represented by analyzing seed to voxel connectivity of the posterior cingulate cortex. Results of all analyses were thresholded at the voxel-level at puncorrected <0.001 and then corrected at the cluster-level using a false-discovery rate (FDR) of p<0.05. (DOCX) [file pone.0134936.s011.docx]

**S3 Table. Default Mode Network Connectivity in Controls**

|  |  |  | MNI Coordinates | | |  |
| --- | --- | --- | --- | --- | --- | --- |
| Region | BA | Cluster Size | x | y | z | *t*-Value |
| Right Frontal Pole | 10 | 12794 | 0 | 64 | 4 | 16.20 |
| Precuneus Cortex | 31 | 6474 | 2 | -54 | 16 | 51.40 |
| Left Middle Temporal Gyrus | 21 | 2963 | -62 | -8 | -20 | 16.03 |
| Left Lateral Occipital Cortex | 19 | 2807 | -40 | -70 | 38 | 13.57 |
| Right Lateral Occipital Cortex | 39 | 2315 | 56 | -62 | 28 | 10.02 |
| Right Temporal Pole | 21 | 1758 | 64 | -2 | -16 | 13.50 |
| Left Hippocampus | 20 | 1526 | -28 | -16 | -18 | 9.83 |
| Right Hippocampus | 35 | 1085 | 22 | -10 | -18 | 8.64 |
| Right Cerebellum | N/A | 467 | -6 | -48 | -38 | 7.43 |
| Right Frontal Pole | 47 | 180 | 38 | 34 | -12 | 8.30 |
| Brain Stem | N/A | 135 | 2 | -24 | -24 | 3.73 |
| Left Crus2 of the Cerebellum | N/A | 99 | -12 | -84 | -40 | 5.72 |
| *Right Frontal Operculum Cortex | 48 | 7704 | 44 | 14 | 2 | 12 |
| *Left Frontal Operculum Cortex | N/A | 5002 | -46 | 10 | 4 | 9.65 |
| * Right VI of the Cerebellum | N/A | 2854 | 38 | -44 | -32 | 6.84 |
| *Left Supramarginal Gyrus | 2 | 1633 | 56 | -34 | 32 | 7.69 |
| *Left Supramarginal Gyrus | 40 | 842 | -60 | -40 | 34 | 7.25 |
| *Right Caudate | N/A | 619 | 18 | -14 | 22 | 8.26 |
| *Left Occipital Pole | 18 | 567 | -14 | -90 | 24 | 4.94 |
| *Right Precentral Gyrus | 6 | 507 | 14 | -28 | 42 | 7.15 |
| *Right Occipital Pole | 18 | 283 | 18 | -92 | 32 | 5.48 |
| *Right VIIIb of the Cerebellum | N/A | 145 | 24 | -42 | -50 | 4.62 |
| *Left VIIIa of the Cerebellum | ? | 107 | -32 | -44 | -50 | 5.68 |

*Note:* ***** denotes negative correlation, otherwise positive correlations are indicated. Connectivity involving the default mode network was represented by analyzing seed to voxel connectivity of the posterior cingulate cortex. Results of all analyses were thresholded at the voxel-level at p_uncorrected_ <0.001 and then corrected at the cluster-level using a false-discovery rate (FDR) of p<0.05
