# Supplementary material for: Intrinsic Functional Connectivity in Salience and Default Mode Networks and Aberrant Social Processes in Youth at Ultra-High Risk for Psychosis
Source: PLoS One. 2015 Aug 7;10(8):e0134936. doi: 10.1371/journal.pone.0134936 (PMC4529226; doi:10.1371/journal.pone.0134936)
Supplement: S4 Table — Note: * denotes negative correlation, otherwise positive correlations are indicated. Connectivity involving the default mode network was represented by analyzing seed to voxel connectivity of the posterior cingulate cortex. Results of all analyses were thresholded at the voxel-level at puncorrected <0.001 and then corrected at the cluster-level using a false-discovery rate (FDR) of p<0.05. (DOCX) [file pone.0134936.s012.docx]

**S4 Table. Default Mode Network Connectivity in UHR**

|  |  |  | MNI Coordinates | | |  |
| --- | --- | --- | --- | --- | --- | --- |
| Region | BA | Cluster Size | x | y | z | *t*-Value |
| Left Frontal Pole | 11 | 15689 | 6 | 48 | -12 | 13.63 |
| Precuneus Cortex | 23 | 10312 | 2 | -56 | 20 | 51.92 |
| Right Middle Temporal Gyrus | 21 | 2872 | 60 | -2 | -20 | 11.36 |
| Left Middle Temporal Gyrus | 21 | 2849 | -58 | -8 | -18 | 11.29 |
| Left Lateral Occipital Cortex | 19 | 2615 | -42 | -68 | 36 | 17.21 |
| Right Lateral Occipital Cortex | 39 | 2356 | 50 | -62 | 30 | 17.09 |
| Right Cerebellum | N/A | 717 | 6 | -48 | -44 | 9.09 |
| Left Crus 2 of the Cerebellum | N/A | 264 | -24 | -80 | -30 | 5.30 |
| Right Frontal Pole | 47 | 206 | 42 | 36 | -12 | 5.14 |
| *Right Central Opercular Cortex | 48 | 5163 | 48 | 8 | 2 | 16.02 |
| *Left Central Opercular Cortex | 48 | 4588 | -46 | 4 | 4 | 12.32 |
| *Right Supplementary Motor Cortex | 6 | 3440 | 8 | -2 | 66 | 8.63 |
| *Right Parietal Operculum Cortex | 48 | 2610 | 62 | -30 | 24 | 9.35 |
| *Left Parietal Operculum Cortex | 48 | 1521 | -56 | -34 | 26 | 7.79 |
| *Left VI of the Cerebellum | N/A | 1090 | -38 | -48 | -32 | 6.59 |
| *Vermis VIIIa of the Cerebellum | N/A | 557 | 4 | -72 | -42 | 5.63 |
| *Right Crus I of the Cerebellum | N/A | 463 | 34 | -70 | -24 | 5.37 |
| *Right VIIIa of the Cerebellum | N/A | 207 | 34 | -44 | -50 | 5.76 |
| *Left Temporal Cortex | 48 | 157 | -26 | -36 | 12 | 5.95 |

*Note:* ***** denotes negative correlation, otherwise positive correlations are indicated. Connectivity involving the default mode network was represented by analyzing seed to voxel connectivity of the posterior cingulate cortex. Results of all analyses were thresholded at the voxel-level at p_uncorrected_ <0.001 and then corrected at the cluster-level using a false-discovery rate (FDR) of p<0.05
